# Note from M. W. Wood, M.D.

**Published:** 1873-10-15

**Authors:** M. W. Wood

**Affiliations:** 1375 State street


Ed. Chi. Med. Examiner: *Sir:*—Since the publication of your journal for Sept. 15th, 1873, I have learned that in 123 cases of Asiatic Cholera, treated by calomel in large doses, in the hands of Surg. J. B. Brown, U. S. A., during the epidemic of 1866, there were but 41 deaths, a mortality of but 33 per cent.; and, as the average mortality of cases without treatment is 50 per cent., justice demands that this more favorable showing of this plan of treatment be stated.

1375 State street. M. W. WOOD, M.D.

